# The mono-ADP-ribosyltransferase ARTD10 regulates the voltage-gated K^+^ channel Kv1.1 through protein kinase C delta

**DOI:** 10.1186/s12915-020-00878-1

**Published:** 2020-10-15

**Authors:** Yuemin Tian, Patricia Korn, Priyanka Tripathi, Daniel Komnig, Dominik Wiemuth, Azadeh Nikouee, Arno Classen, Carsten Bolm, Björn H. Falkenburger, Bernhard Lüscher, Stefan Gründer

**Affiliations:** 1 Institute of Physiology, RWTH Aachen University, Pauwelsstrasse 30, 52074 Aachen, Germany; 2 Institute of Biochemistry and Molecular Biology, RWTH Aachen University, Pauwelsstrasse 30, 52074 Aachen, Germany; 3 Present address: Institute of Neuropathology, RWTH Aachen University Medical School, Pauwelsstrasse 30, 52074 Aachen, Germany; 4 Department of Neurology, RWTH Aachen University, Pauwelsstrasse 30, 52074 Aachen, Germany; 5 JARA-Institute Molecular Neuroscience and Neuroimaging, Forschungszentrum Jülich, Jülich, Germany; 6 Institute of Organic Chemistry, RWTH Aachen University, Landoltweg 1, 52056 Aachen, Germany; 7 Present address: Department of Neurology, Dresden University Medical Center, Fetscherstraße 74, 01307 Dresden, Germany

**Keywords:** Ion channel, Potassium channel, ADP ribosylation, Posttranslational modification, ADP ribosyltransferase, Protein kinase C delta, PARP10

## Abstract

**Background:**

ADP-ribosylation is a ubiquitous post-translational modification that involves both mono- and poly-ADP-ribosylation. ARTD10, also known as PARP10, mediates mono-ADP-ribosylation (MARylation) of substrate proteins. A previous screen identified protein kinase C delta (PKCδ) as a potential ARTD10 substrate, among several other kinases. The voltage-gated K^+^ channel Kv1.1 constitutes one of the dominant Kv channels in neurons of the central nervous system and the inactivation properties of Kv1.1 are modulated by PKC. In this study, we addressed the role of ARTD10-PKCδ as a regulator of Kv1.1.

**Results:**

We found that ARTD10 inhibited PKCδ, which increased Kv1.1 current amplitude and the proportion of the inactivating current component in HeLa cells, indicating that ARTD10 regulates Kv1.1 in living cells. An inhibitor of ARTD10, OUL35, significantly decreased peak amplitude together with the proportion of the inactivating current component of Kv1.1-containing channels in primary hippocampal neurons, demonstrating that the ARTD10-PKCδ signaling cascade regulates native Kv1.1. Moreover, we show that the pharmacological blockade of ARTD10 increases excitability of hippocampal neurons.

**Conclusions:**

Our results, for the first time, suggest that MARylation by ARTD10 controls neuronal excitability.

## Background

ADP-ribosyltransferases (ARTs) regulate many cellular processes including DNA damage repair and transcriptional regulation by post-translational modification of their target proteins with ADP-ribose groups [1]. Diphtheria toxin-like ADP-ribosyltransferases are classified as ARTDs [2]. While ARTD1, also named PARP1 and the founding member of the PARP family, transfers iteratively several ADP-ribose groups to its target proteins in a process termed poly-ADP-ribosylation (or PARylation) [3], several other ARTDs, including ARTD10, transfer only a single ADP-ribose group onto their target proteins (mono-ADP-ribosylation or MARylation) [4]. MARylation is a widely used posttranslational modification; however, only few cellular substrates have been identified and the functional consequences are ill defined.

ARTD10 was initially described as an interaction partner of the nuclear oncoprotein MYC [5]. ARTD10 MARylates itself and core histones [4]. It shuttles between the nucleus and the cytosol, but resides mainly in the cytosol [6], suggesting that it also has cytoplasmic substrates. Protein microarrays revealed several potential substrates of ARTD10, 32% of which were kinases [7]. For one of them, glycogen synthase kinase 3β (GSK3β), it was verified that it is MARylated and that this modification reversibly inhibits its enzymatic function [7, 8].

In the current study, we confirmed MARylation of another potential ARTD10 substrate [7], protein kinase Cδ (PKCδ). We found that MARylation reduced catalytic activity of PKCδ, similar to GSK3β. To elucidate the functional effect of PKCδ MARylation, we used the modulation of an important voltage-gated K^+^ channel, Kv1.1, by PKC.

In the nervous system, voltage-gated K^+^ (Kv) channels play a critical role in action potential (AP) initiation and propagation, and in the regulation of spike patterns [9]. The Kv1.1 subunits form heteromeric channel complexes with other members of the Kv1-family (mainly with Kv1.2/1.6/1.4) and auxiliary β subunits [10]. Native heterooligomeric complexes form one of the dominant Kv channels in central axons, and they also affect somato-dendritic excitability in some neurons, prominently among them hippocampal CA1 neurons where Kv1 channels mediate the D-type K^+^ current (“delay current”) [11]. The importance of Kv1.1 is underlined by the fact that mutations in the *KCNA1* gene, coding for the α subunit of Kv1.1, lead to episodic ataxia with myokymia (rippling of muscles) [12]. Moreover, mice with a genetic Kv1.1 knock-out exhibit hippocampal and peripheral nerve hyper-excitability and severe epilepsy [13]. Similarly, autosomal dominant epilepsy with auditory features also results from a reduced density of axonal Kv1.1 channels in hippocampal CA3 neurons [14]. Thus, a better knowledge of the mechanisms of Kv1.1 regulation is important for a better understanding of network plasticity and neuronal hyperexcitability disorders.

Regulation of Kv1.1 channels is complex given that they are composed of four pore-forming α and four β subunits [10, 15]. Association with a cytoplasmic β subunit (Kvβ1) confers inactivation to the channel complex [16]. Regulatory mechanisms thus affect both the α and the β subunits, often in a complex interplay [17–20]. For example, the inactivation conferred by Kvβ1 is controlled by phosphorylation of the α subunit at serine-446 (S446) by protein kinase A (PKA) [21–23]. PKC, in contrast, does not phosphorylate Kv1.1 directly [22] but indirectly causes the de-phosphorylation of the α subunit at S446 [24], thus counter-acting the effect of PKA and reducing the proportion of inactivating Kv1.1 currents. We hypothesized that ARTD10 through its effects on PKCδ indirectly modulates inactivation of Kv1.1, thereby modulating excitability of neurons.

## Results

### Mono-ADP-ribosylation by ARTD10 reduces PKCδ activity

**Fig. 1:**
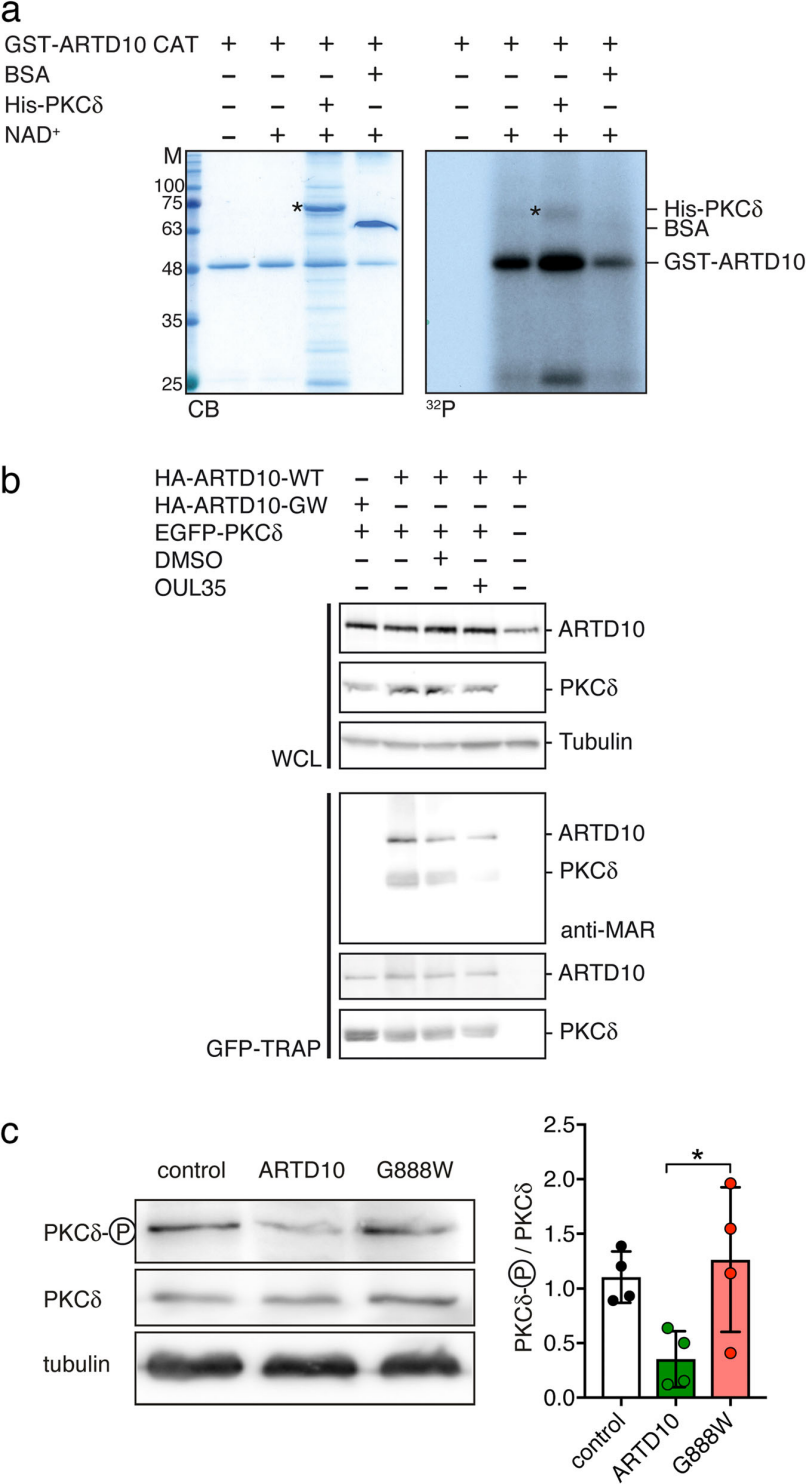
MARylation of PKCδ by ARTD10 reduces its kinase activity. **a** In vitro ADP-ribosylation assay of the GST-ARTD10 catalytic domain (818-1025) with His_6_-PKCδ with or without [^32^P]-γ-NAD^+^. BSA was included as substrate negative control. Exposure to X-Ray (^32^P) was allowed for 96 h. CB, Coomassie blue; ^32^P, autoradiogram. **b** Western blot analysis of MARylation of immunoprecipitated PKCδ from HEK293 cells transiently expressing exogenous PKCδ in the presence of ARTD10 or ARTD10-G888W. The expression of the proteins was verified in whole cell lysates (WCL). Treatment with OUL35 or vehicle control (DMSO) was as indicated. **c** Left, Western blots of phosphorylated PKCδ (Y311), indicating active PKCδ, and of total PKCδ from control HeLa cells or HeLa cells expressing wild type ARTD10 or ARTD10-G888W. Right, ratio of phosphorylated PKCδ/total PKCδ (*n* = 4). *p < 0.05 (one-way ANOVA followed by Tukey’s test)

To confirm PKCδ as a substrate of ARTD10, we performed in vitro ADP-ribosylation assays with the purified catalytic domain of ARTD10 fused to GST (GST-ARTD10 CAT) and of PKCδ fused to a 6-His-tag (His-PKCδ; Fig. 1a). As expected, ARTD10 MARylated itself. In addition, PKCδ was also MARylated, while BSA as a negative control was not. Although MARylation of PKCδ was less effective than auto-MARylation of ARTD10 (Fig. 1a), this result confirms PKCδ as a substrate of ARTD10 in vitro.

Next, we analyzed PKCδ MARylation in cells. GFP-PKCδ and HA-ARTD10 or a catalytically inactive mutant (HA-ARTD10-G888W) [25] were transiently expressed in HEK293 cells, GFP-PKCδ was enriched using a GFP-TRAP, and MARylation was analyzed by immunoblotting. PKCδ was MARylated in the presence of ARTD10 but not when PKCδ was expressed alone or in the presence of ARTD10-G888W (Fig. 1b). Moreover, MARylation of PKCδ when co-expressed with ARTD10 was reduced in the presence of the selective ARTD10 inhibitor OUL35 [26]. These results demonstrate MARylation of PKCδ by ARTD10 in living cells. Further, ARTD10 as well as ARTD10-G888W were co-immunoprecipitated with PKCδ using the GFP-TRAP from lysates (Fig. 1b). Thus, this interaction was independent of catalytic activity of ARTD10. In the absence of PKCδ no ARTD10 was trapped, indicating that the interaction was specific.

HeLa cells express the classical PKC isoform PKCα, the atypical isoform PKCζ, as well as the novel isoform PKCδ [27]. Phosphorylation at tyrosine 311 (Y311) between the regulatory and catalytic domains of PKCδ is a critical step for its activation [28] and can be used as a read-out of PKCδ activity. To further corroborate MARylation of PKCδ in living cells, we therefore used HeLa cells stably expressing either wildtype (WT) ARTD10 or mutant ARTD10-G888W [25] and compared the phosphorylation status of PKCδ in these cells with that of control HeLa cells. While the total abundance of PKCδ was similar in all three cell lines, the abundance of PKCδ phosphorylated at Y311 was reduced in ARTD10-WT expressing cells compared with control HeLa cells or cells expressing ARTD10-G888W (Fig. 1c). This finding indicates that MARylation of PKCδ causes dephosphorylation of PKCδ. It is reminiscent of the ARTD10 effect on GSK3β, which leads to decreased phosphorylation and decreased kinase activity [7]. Given that phosphorylation at Y311 activates PKCδ [28], our results therefore suggest that MARylation of PKCδ by ARTD10 reduces its catalytic activity.

### PKCδ reduces the proportion of inactivating Kv1.1 currents via dephosphorylation at S446 of the α subunit

**Fig. 2:**
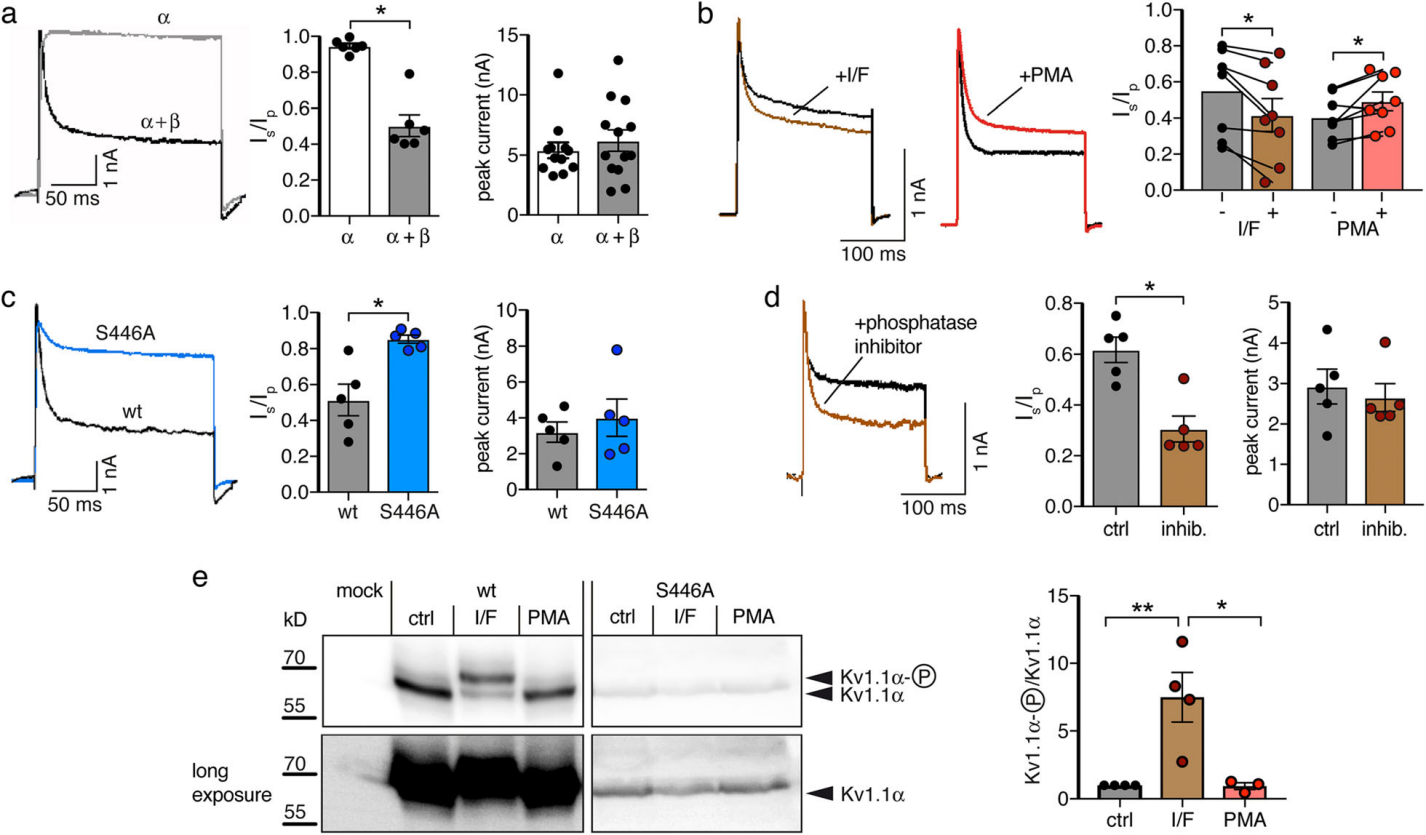
Inactivation of Kv1.1 is regulated by Kvβ and phosphorylation at S446. **a** Representative recordings, *I*_steady-state_/*I*_peak_ (*I*_s_/*I*_p_), and peak current amplitudes of Kv1.1 with or without co-expression of Kvβ1.1 in HeLa cells. **b** Representative recordings and *I*_s_/*I*_p_ before and after the application of either IBMX/forskolin (I/F) or of the phorbol ester PMA. **c** Representative recordings, *I*_s_/*I*_p_, and peak current amplitudes of the phosphorylation-deficient mutant Kv1.1_S446A_ co-expressed with Kvβ1.1. **d** Representative recordings, *I*_s_/*I*_p_, and peak current amplitudes of Kv1.1 after 1.5 h pre-incubation with a phosphatase inhibitor cocktail. *p < 0.05 (Student’s *t* test). **e** Left, Western blot with an anti-Kv1.1α antibody revealing phosphorylated and unphosphorylated Kv1.1α. At the bottom a longer exposure of the same blot is shown to demonstrate loss-of-phosphorylation when S446 was substituted by A. Protein levels of Kv1.1-S446A were consistently lower than of wild-type Kv1.1. Right, ratio of phosphorylated and unphosphorylated Kv1.1α, normalized to control conditions (*n* = 4 for control and I/F, *n* = 3 for PMA)

To investigate the modulation of the voltage-gated K^+^ channel Kv1.1 by PKC in HeLa cells, we transiently expressed Kv1.1 in HeLa cells and evoked K^+^ currents by a depolarization from − 80 mV to + 40 mV. Transient expression of Kv1.1α alone induced robust non-inactivating K^+^ currents; additional co-expression of the β1.1 subunit conferred fast and partial inactivation to these currents. We quantified the proportion of the non-inactivating current component by building the ratio of the current amplitude at the end of a 200-ms pulse to +40 mV (*I*_steady-state_) to the peak current at the beginning of this pulse (*I*_peak_; Fig. 2a). Inactivation was best fit with a bi-exponential function, yielding a fast *τ*_1_ = 7.2 ± 0.3 ms and a slow *τ*_2_ = 82.9 ± 8.1 ms (*n* = 6). Peak currents were similar with and without the β subunit (Fig. 2a). In cells co-expressing Kv1.1α and Kvβ1.1, 5 min application of IBMX and forskolin, to increase cAMP levels and activate PKA, decreased the proportion of the non-inactivating K^+^ current (*I*_steady-state_/*I*_peak_, from 0.55 ± 0.08 to 0.42 ± 0.09, *n* = 8, *p* = 0.005, paired Student’s *t* test), while 5-min application of the phorbol ester phorbol 12-myristate 13-acetate (PMA) to activate PKC increased it (*I*_steady-state_/*I*_peak_, from 0.40 ± 0.04 to 0.49 ± 0.05, *n* = 8, *p* = 0.022, paired Student’s *t* test; Fig. 2b). This is in agreement with previous reports [21]. To confirm the importance of phosphorylation at S446 for the inactivation, we co-expressed the phosphorylation-deficient mutant Kv1.1α-S446A together with Kvβ1.1. Compared to wild-type Kv1.1α, the proportion of the non-inactivating component was strongly increased for Kv1.1α-S446A (*I*_steady-state_/*I*_peak_, 0.85 ± 0.02 vs. 0.51 ± 0.09, *n* = 5, *p* = 0.006; Fig. 2c), although the β subunit binds equally well to Kv1.1α-S446A and to wild-type Kv1.1α [23]. These results confirm that phosphorylation of S446 promotes inactivation [23]. Peak current amplitudes were similar (Fig. 2c). Moreover, a phosphatase inhibitor cocktail strongly decreased the proportion of non-inactivating K^+^ currents in HeLa cells expressing Kv1.1 (*I*_steady-state_/*I*_peak_, 0.31 ± 0.05 vs. 0.62 ± 0.05, *n* = 5, *p* = 0.002; Fig. 2d), suggesting that phosphorylation/dephosphorylation of S446 is dynamic in these cells.

To directly show enhanced Kv1.1 phosphorylation after stimulation of PKA, we analyzed Kv1.1α by Western blotting. It has been reported that the phosphorylated form of Kv1.1 migrates at 57 kD and the un-phosphorylated form at 54 kD [21, 23]. After expression in HeLa cells, two bands with an apparent molecular weight of approximately 60 kDa were observed (Fig. 2e). The ratio between the higher band, corresponding to phosphorylated Kv1.1α, and the lower band, corresponding to un-phosphorylated Kv1.1α, was strongly increased in HeLa cells stimulated with IBMX and forskolin compared to control cells (Fig. 2e). PMA application only slightly reduced phosphorylation. In contrast, in cells expressing the phosphorylation-site mutant Kv1.1-S446A, only the smaller band, corresponding to un-phosphorylated Kv1.1α, was observed (Fig. 2e). These results confirm the phosphorylation of Kv1.1α at S446 by PKA.

**Fig. 3:**
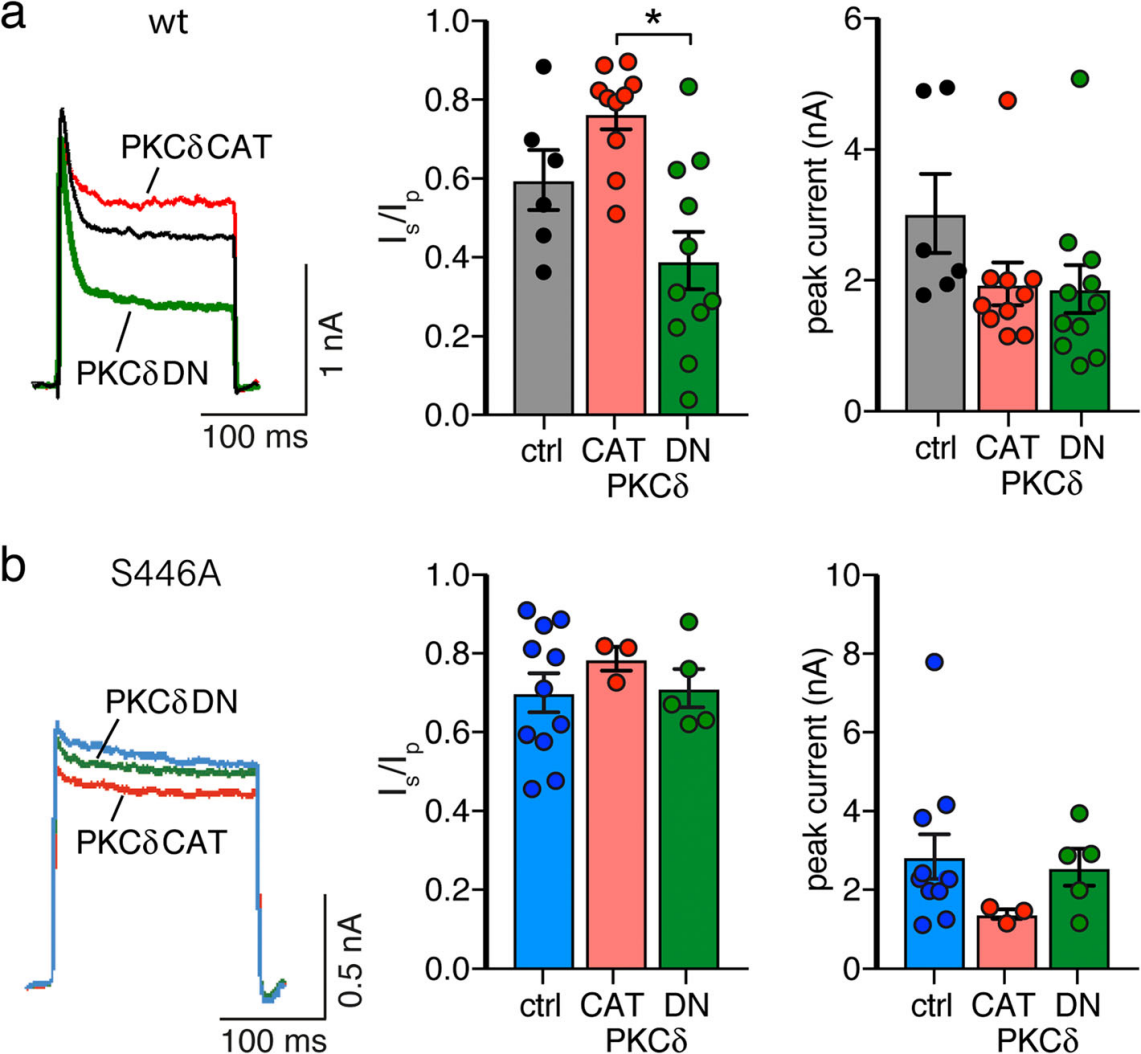
Kv1.1 is regulated by PKCδ in HeLa cells. **a**, **b** Wild type Kv1.1 or mutant Kv1.1-S446A were co-expressed with Kvβ1 and either the catalytic domain (CAT) or a dominant negative mutant (DN) of PKCδ. Bar graphs represent *I*_steady-state_/*I*_peak_ (*I*_s_/*I*_p_) and peak current amplitudes

In HeLa cells, phorbol esters activate PKCδ and PKCα, but not the atypical isoform PKCζ [27]. To confirm that PKCδ is the subtype of PKC, which was responsible for the reduced extent of inactivation after PMA treatment, we co-expressed Kv1.1 together either with a constitutively active catalytic domain of PKCδ (PKCδ-CAT), or with a dominant negative PKCδ (PKCδ-DN) [29]. Cells co-expressing PKCδ-CAT had a large non-inactivating current component while cells co-expressing PKCδ-DN had a small non-inactivating component (*I*_steady-state_/*I*_peak_, PKCδ-CAT = 0.77 ± 0.04, vs. PKCδ-DN = 0.39 ± 0.07; *n* = 10 for PKCδ-CAT, *n* = 11 for PKCδ-DN, *p* < 0.001; Fig. 3a), in agreement with a modulation of the extent of inactivation by PKCδ. The peak current amplitude was not significantly different between both groups (PKCδ-CAT = 1.94 ± 0.31 nA vs. PKCδ-DN = 1.86 ± 0.35 nA *p* = 0.99; Fig. 3a). When the phosphorylation-deficient mutant Kv1.1-S446A was co-expressed with either PKCδ-CAT or PKCδ-DN, both groups of cells had a similarly large proportion of non-inactivating currents (Fig. 3b), confirming that PKCδ reduces inactivation of Kv1.1 via inducing dephosphorylation of Kv1.1α at S446 in HeLa cells.

### ARTD10 increases the inactivating current component of Kv1.1 via phosphorylation at S446

**Fig. 4:**
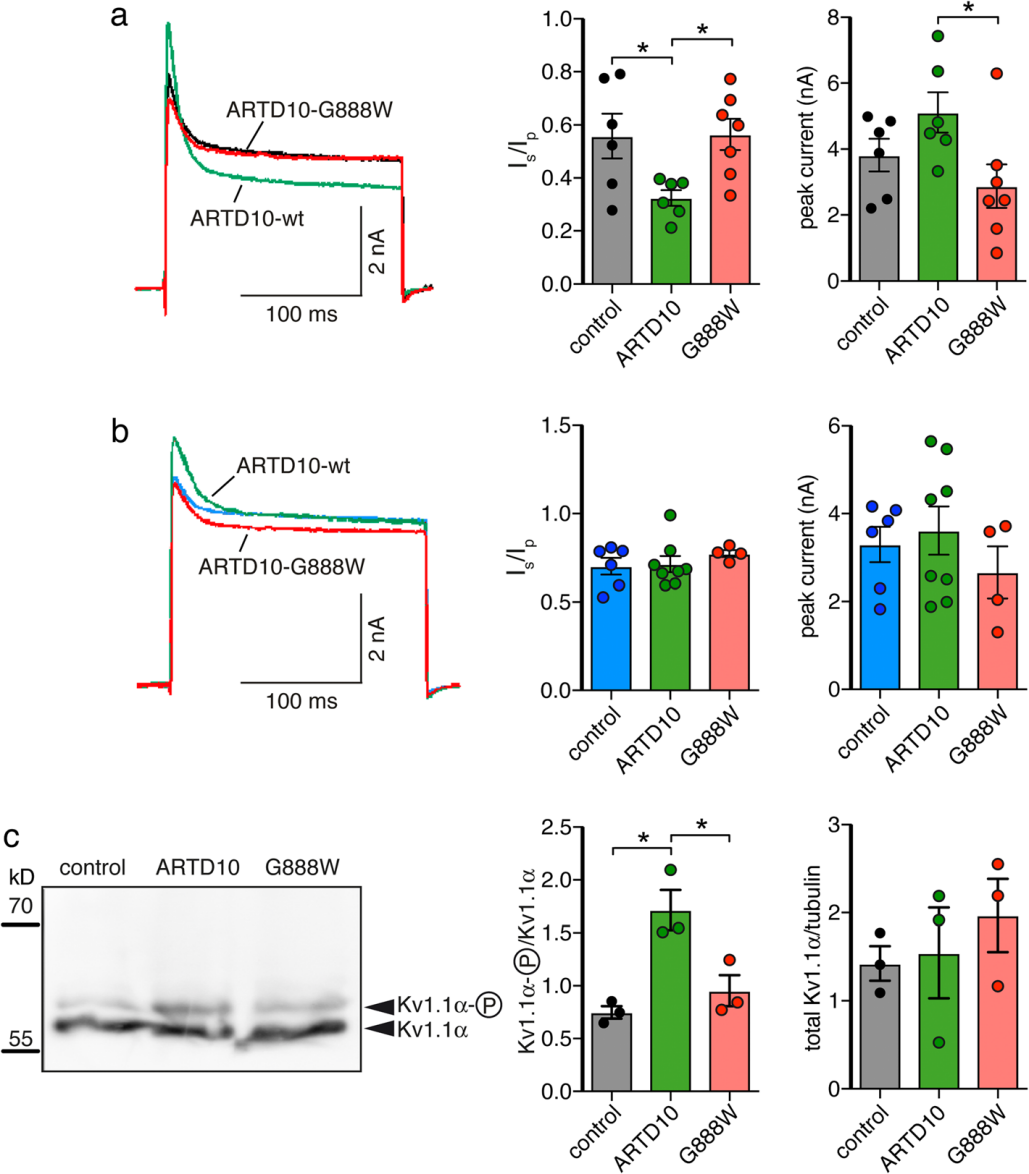
ARTD10 leads to phosphorylation of Kv1.1 at S446. **a**, **b** Left, Representative current traces of wild type (**a**) or the phosphorylation-deficient mutant (**b**) of Kv1.1 overexpressed in three different HeLa cell lines. Right, bar graphs representing *I*_steady-state_/*I*_peak_ (*I*_s_/*I*_p_) and peak current amplitudes. *p < 0.05 (one-way ANOVA followed by Tukey’s test). **c** Left, total Kv1.1α protein from control HeLa cells or from stably transfected HeLa cells expressing wild type or catalytically inactive ARTD10. Right, ratio of phosphorylated and unphosphorylated Kv1.1α and of total Kv1.1α and tubulin (*n* = 3)

To investigate the effect of PKCδ MARylation on Kv1.1, we transiently expressed Kv1.1 in control HeLa cells, in HeLa cells stably expressing ARTD10-WT and in HeLa cells stably expressing ARTD10-G888W. The non-inactivating current component of Kv1.1 was significantly smaller in cells expressing ARTD10-WT than in either control cells or cells expressing ARTD10-G888W (*I*_steady-state_/*I*_peak_, ARTD10 = 0.32 ± 0.03 vs. control = 0.56 ± 0.08, *n* = 6, and ARTD10-G888W = 0.56 ± 0.06, *n* = 7, *p* = 0.04; Fig. 4a). This is consistent with a stronger phosphorylation of the Kv1.1α at S446 and thus with a decreased PKCδ activity in ARTD10-expressing cells. Moreover, there was a tendency for a larger peak current amplitude in ARTD10-expressing cells than in control cells (5.12 ± 0.61 nA vs. 3.82 ± 0.49 nA, *n* = 6, *p* = 0.13); amplitudes of Kv currents in ARTD10-G888W-expressing cells were significantly smaller than in cells expressing ARTD10 wild-type (2.88 ± 0.66 nA, *n* = 7, *p* = 0.03) (Fig. 4a). These results suggest increased Kv1.1 activity after phosphorylation at S446, as has previously been observed [23]. When the phosphorylation-deficient mutant Kv1.1-S446A was expressed, no difference between groups was observed (Fig. 4b), demonstrating that phosphorylation at S446 was indeed important for the modulation of Kv1.1 inactivation by ARTD10. This observation also supports the conclusion that the ARTD10 effect is mediated by PKCδ.

To directly show enhanced Kv1.1 phosphorylation in ARTD10-expressing cells, we analyzed Kv1.1α by Western blot. In ARTD10-expressing HeLa cells, the ratio between the higher band, corresponding to phosphorylated Kv1.1, and the lower band, corresponding to un-phosphorylated Kv1.1, was significantly larger compared to control or to ARTD10-G888W-expressing cells (Fig. 4c), directly demonstrating that the presence of ARTD10 increases phosphorylation of Kv1.1.

### An ARTD10 inhibitor decreases inactivation of K^+^ currents and increases excitability of hippocampal neurons

**Fig. 5:**
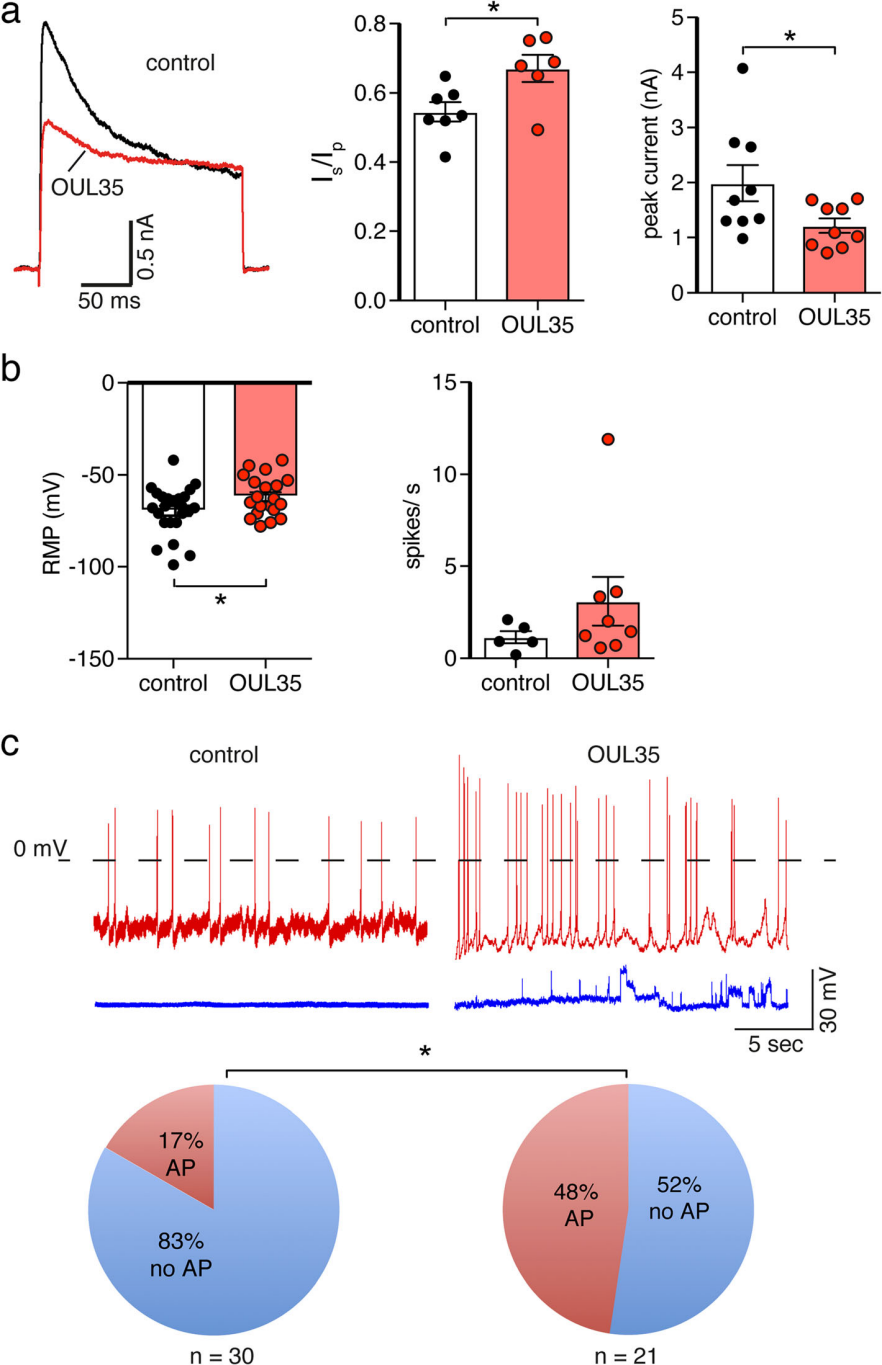
ARTD10 inhibition reduces the proportion of the inactivating Kv1.1 current and enhances spontaneous excitation in hippocampal neurons. **a** Left, whole cell currents of mouse hippocampal neurons in the presence of tetrodotoxin with and without an inhibitor of ARTD10 (OUL35). Right, bar graphs representing *I*_steady-state_/*I*_peak_ (*I*_s_/*I*_p_) and peak current amplitudes. **b** Resting membrane potential (RMP) and spontaneous spikes/s from control and OUL35 treated hippocampal neurons. *p < 0.05 (Student’s *t* test). **c** Top, examples of current clamp recordings of control (left) and OUL35 treated hippocampal neurons (right). Some of the cells had spontaneous APs (red), and some not (blue). Bottom, pie chart illustrating the proportion of cells with or without spontaneous APs. *p < 0.05 (Fisher’s exact test)

Kv1.1 together with other Kv1α and Kvβ subunits are widely expressed in the brain [10]. Kv1 channels have been extensively studied in hippocampal CA1 neurons, where they mediate the D-type K^+^ current (“delay current”) [11, 30]. Although different Kv1α subunits may contribute to the D current, it has been reported that in hippocampal neurons cultivated for 6–11 days in vitro (DIV) Kv1.1 is the only Kv1 protein expressed [31]. ARTD10 is expressed at low levels in many brain regions, including hippocampus. PKCδ has a relatively low expression in mouse hippocampus at postnatal days 2–6, but its expression increases strongly afterwards [32]. We, therefore, focused on hippocampal neurons to investigate the modulation of Kv1 channel function and neuronal excitability by ARTD10. We treated isolated hippocampal neurons in culture with the selective inhibitor of ARTD10, OUL35 (3 μM) [26], at DIV 7 and performed patch clamp recordings after 3 to 5 days (DIV 10-12). K^+^ currents were evoked by depolarizing neurons from − 80 mV to + 40 mV. K^+^ currents elicited in hippocampal neurons resembled Kv1.1 currents recorded in HeLa cells, but inactivated slightly more slowly (*τ*_1_ = 17.8 ± 1.4 ms, *τ*_2_ = 175.5 ± 35.5 ms, *n* = 7). Strikingly, voltage-gated K^+^ currents in neurons incubated with OUL35 had significantly decreased peak amplitudes (1.22 ± 0.13 nA, *n* = 6, vs. 1.99 ± 0.33 nA, *n* = 7, *p* = 0.04) and an increased proportion of non-inactivating currents (*I*_steady-state_/*I*_peak_, OUL35 = 0.67 ± 0.04 vs. DMSO control = 0.55 ± 0.03, *p* = 0.02; Fig. 5a), which is expected if inhibition of ARTD10 would increase PKCδ activity in these neurons and concomitantly reduce phosphorylation at S446 of Kv1.1. Thus, voltage-clamp experiments suggested that the ARTD10-inhibitor OUL35 affected the D-current in hippocampal neurons.

We then assessed the intrinsic excitability of cultured hippocampal neurons recorded under current clamp in a perforated patch configuration. First, we noticed that neurons treated with OUL35 had a significantly depolarized membrane potential as compared to untreated neurons (− 61.8 ± 2.4 mV vs. − 69.5 ± 2.7 mV, *p* = 0.04; Fig. 5b left). Although Kv1.1 is not expected to strongly control the resting membrane potential, it has previously been reported that PKA phosphorylation of Kv1.1 negatively shifts the resting membrane potential [22]. The positive shift of the membrane potential that we observed in OUL35 treated neurons would thus be compatible with a reduced phosphorylation of Kv1.1. The slightly depolarized membrane potential is expected to bring neurons closer to the threshold potential. Indeed, almost half of the OUL35-treated neurons showed spontaneous action potentials (APs), while only 17% of neurons without treatment showed spontaneous APs (*p* = 0.028, Fisher’s exact test; Fig. 5c). Within the pool of spontaneously active neurons, neurons incubated with OUL35 also had a tendency for an increased AP frequency (3.1 ± 1.3 spikes/s vs. 1.15 ± 0.33 spikes/s, *p* = 0.27; Fig. 5b right). When we excluded strongly depolarized cells with a resting membrane potential more positive than − 60 mV, only 33% of the OUL35-treated neurons showed spontaneous APs (*n* = 15) compared with 15% of the control neurons (*n* = 27; *p* = 0.24, Fisher’s exact test) and there was still a tendency for an increased AP frequency (1.87 ± 0.78 spikes/s vs. 2.91 ± 1.10 spikes/s, *p* = 0.25).

**Fig. 6:**
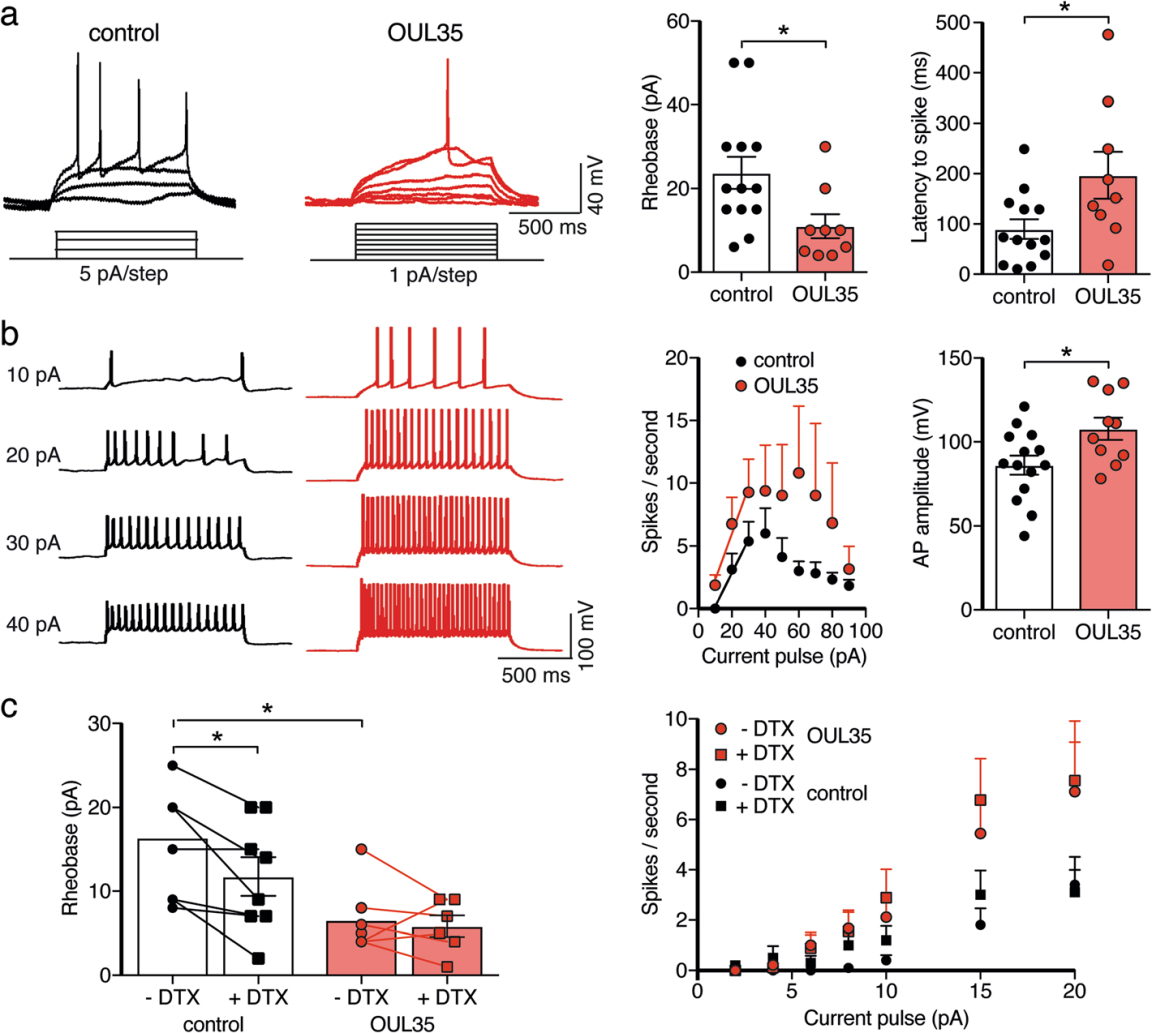
ARTD10 inhibition enhances excitability of hippocampal neurons via Kv1.1. **a** Left, representative current clamp recordings of APs elicited by step current pulses in control neurons and neurons treated with OUL35. Right, bar graphs represent the rheobase and the latency to the first spike. For cells with a RMP more positive than − 60 mV, the membrane potential was adjusted to ~ − 60 mV. **b** The number of spikes elicited by step current pulses were counted and for stimuli from 10 to 30 pA they were fitted with a linear function. Right, bar graphs summarize the AP amplitude from neurons with and without OUL35 treatment. **c** Left, bar graphs representing the rheobase. Right, summary of spikes/s with and without the Kv1 inhibitor α-dendrotoxin (DTX) and from neurons with and without OUL35 treatment. *p < 0.05 (Student’s *t* test)

To further test the excitability of hippocampal neurons, we used current clamp and a step protocol to apply current pulses of gradually increasing amplitude and determined the current that elicited the first AP (rheobase). Neurons incubated with OUL35 for 72 h displayed a robust increase in excitability with a significant decrease in rheobase (11 ± 2.9 pA, *n* = 9, vs. 23.8 ± 3.9 pA, *n* = 13, *p* = 0.02; Fig. 6a). Moreover, counting the numbers of APs generated by current injections of higher amplitude revealed that the neurons treated with OUL35 had an increased AP frequency compared with the control (Fig. 6b). In both groups, AP frequency started to plateau at current pulses of 40 pA and decreased at higher amplitude pulses, probably due to a depolarization block. The amplitude of APs was increased in OUL35-treated neurons (Fig. 6b), which is consistent with a reduced repolarizing current.

To ascertain the importance of Kv1 channels for the increase in excitability of hippocampal neurons by OUL35, we inhibited D-type K^+^ currents with the snake toxin α-dendrotoxin (DTX; 100 nM), a specific blocker of channels containing Kv1.1, Kv1.2 or Kv1.6 subunits [33]. Like previously reported [34], in control neurons, DTX application decreased the rheobase (from 16.38 ± 2.52 pA to 11.75 ± 2.31 pA, *p* = 0.008, paired Student’s *t* test; Fig. 6c left). In contrast, in neurons treated with OUL35, DTX had no effect on the rheobase (5.83 ± 1.27 pA vs. 6.57 ± 1.51 pA, *p* = 0.54; Fig. 6c). OUL35, however, robustly decreased the rheobase (6.57 ± 1.51 pA vs. 16.38 ± 2.52 pA, *p* = 0.006; Fig. 6c), as we observed before (Fig. 6a). These data show that OUL35 occluded DTX-sensitivity of hippocampal neurons, suggesting that OUL35 reduced the activity of native DTX-sensitive Kv1 channels. The stronger reduction in rheobase by OUL35 than by DTX (Fig. 6c) might indicate that OUL35 affected rheobase by Kv1.1 and another unknown mechanism. In addition to the reduced rheobase, there was a tendency of increased AP frequency by DTX-treatment in both control and OUL35 treated group (Fig. 6c).

**Fig. 7:**
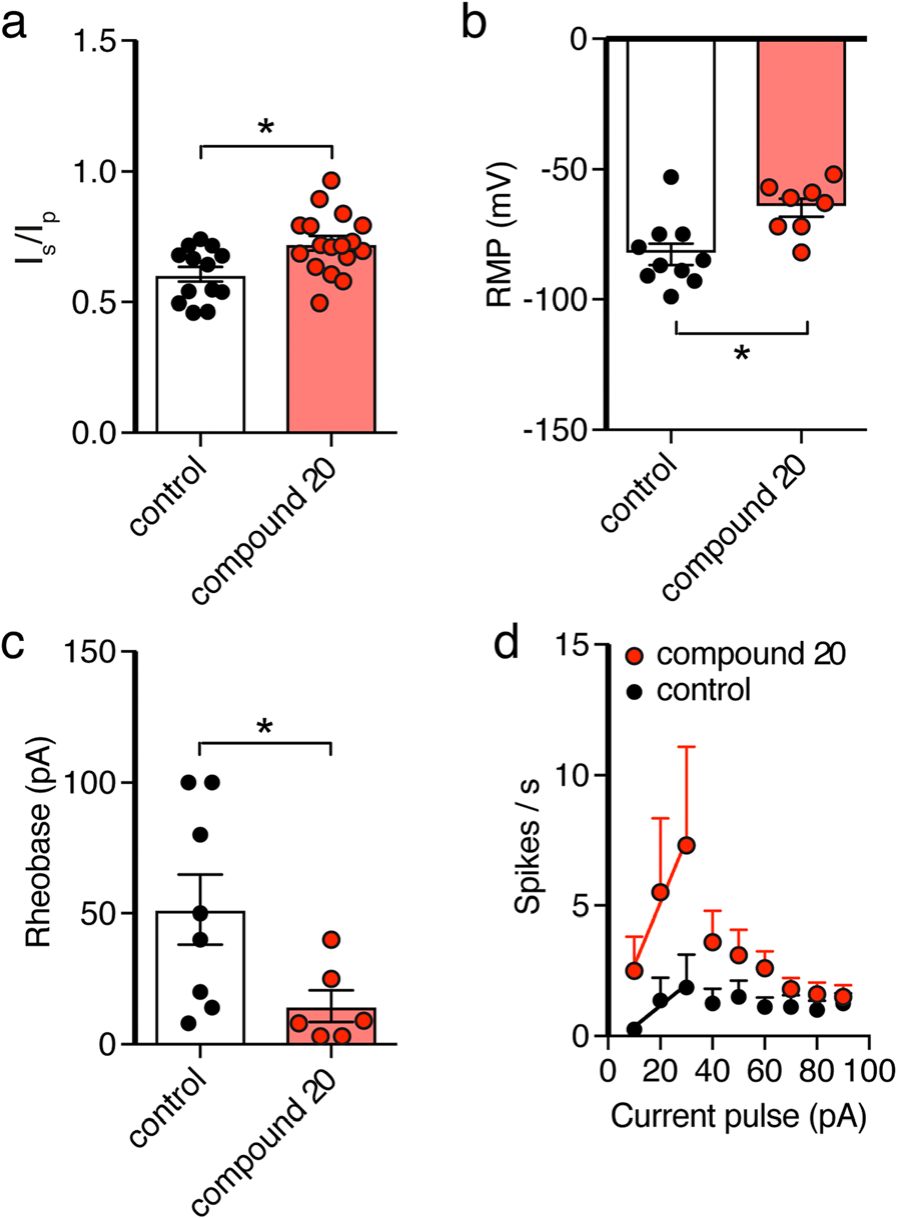
The ARTD10 inhibitor compound 20 has similar effects as OUL35 in hippocampal neurons. Bar graphs representing the effect of compound 20 on *I*_steady-state_/*I*_peak_ (*I*_s_/*I*_p_) (**a**), resting membrane potential (**b**), rheobase (**c**), and spikes/s (**d**). *p < 0.05 (Student’s *t* test)

We confirmed the specific inhibition of ARTD10 by OUL35 in hippocampal neurons with another ARTD10 inhibitor, compound 20 (Fig. 7). This compound, which will be described in a future manuscript, had similar effects on excitability of hippocampal neurons as OUL35: it increased the proportion of the non-inactivating component of K^+^ currents (*I*_steady-state_/*I*_peak_, 0.72 ± 0.03 vs. 0.61 ± 0.03, *p* = 0.007; Fig. 7a), it depolarized the resting membrane potential (− 64.7 ± 3.5 mV vs. − 82.7 ± 4.1 mV, *p* = 0.005; Fig. 7c), it strongly reduced the rheobase (14.6 ± 6 pA vs. 51.5 ± 13.3 pA, *p* = 0.04; Fig. 7b), and it increased the frequency of evoked APs (*p* = 0.007, unpaired Student’s *t* test; Fig. 7d). These results confirm that inhibiting ARTD10 in hippocampal neurons change the inactivation of K^+^ channels and increase neuronal excitability.

## Discussion

**Fig. 8:**
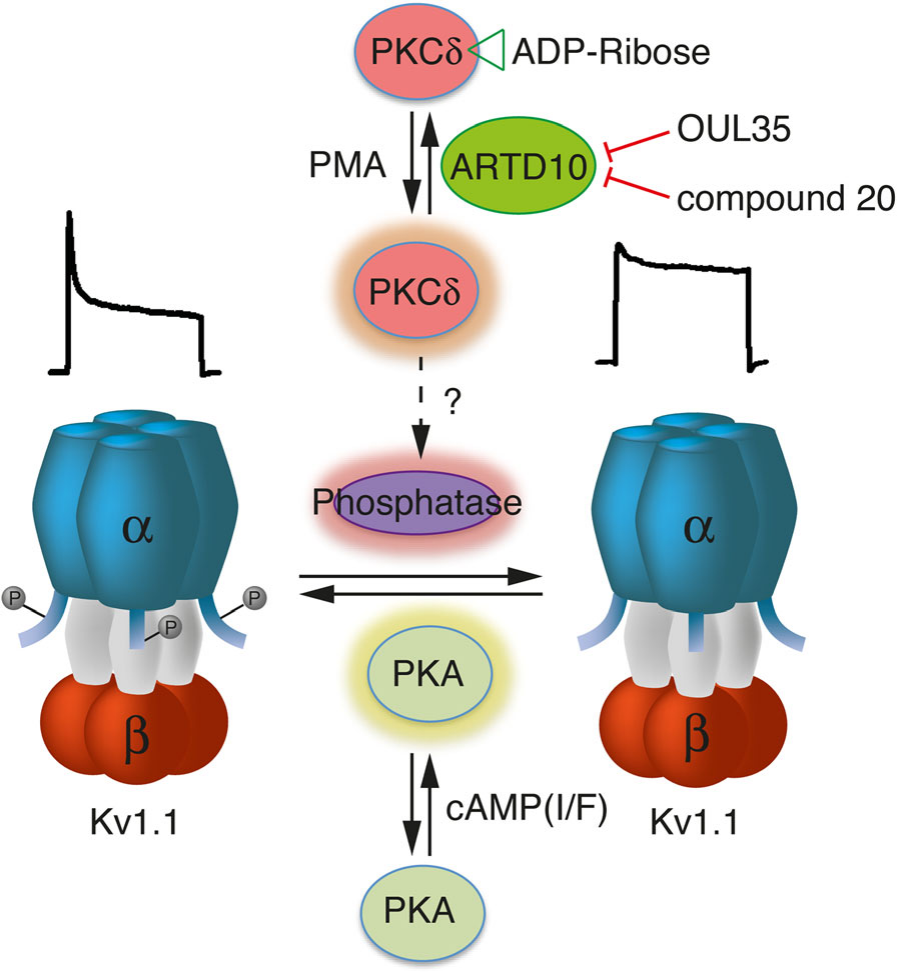
Scheme illustrating the interplay of PKA, PKCδ, and ARTD10 in the regulation of Kv1.1. The regulation of a phosphatase by PKCδ is hypothetical. Current traces above the schemes of the Kv1.1 channels illustrate the typical inactivation pattern of phosphorylated vs. un-phosphorylated Kv1.1α. I/F, IBMX/forskolin; PMA, phorbol-myristate-acetate

In this study, we show (1) that PKCδ is a substrate of ARTD10 in vitro as well as in cells, (2) that co-expression with ARTD10 reduces PKCδ activity, and (3) that co-expression of ARTD10 with Kv1.1 leads to an increased phosphorylation of Kv1.1α on S446, which in turn decreases the non-inactivating current component of Kv1.1. Together, these results suggest that ARTD10 indirectly regulates the gating of Kv1.1 via PKCδ. This regulation is summarized in Fig. 8.

Kv1.1 is one of the main K^+^ channels in the nervous system and regulation of its gating, in particular inactivation, has a major influence on neuronal excitability. Therefore, the regulation of Kv1.1 by ARTD10-PKCδ has the potential to greatly impact on neuronal excitability. As a first step to assess the importance of ARTD10 for neuronal excitability, we analyzed hippocampal neurons, kept for 10–12 days in vitro, and which we treated with the ARTD10-inhibitor OUL35 for 3 days to chronically inhibit ARTD10.

In these hippocampal cultures, outward K^+^ currents closely resembled K^+^ currents in HeLa cells over-expressing Kv1.1 (compare for example Fig. 5a with Fig. 4a). Although endogenous K^+^ channels in hippocampal neurons are likely formed by different Kvα subunits, it has been reported that at DIV 6-11 Kv1.1α makes a substantial contribution in these neurons [31]. We found that OUL35 treatment reduced the peak amplitude of K^+^ currents and increased the non-inactivating current component. Strikingly, these effects are just opposite to what we observed after co-expression of Kv1.1α with ARTD10 in HeLa cells (Fig. 4a). Thus, the effects of OUL35 in hippocampal neurons are consistent with the idea that an inhibition of ARTD10 results in increased PKCδ activity and subsequently an indirect dephosphorylation of Kv1.1α (Fig. 8). We cannot exclude, however, that OUL35 has additional effects in hippocampal neurons.

Together with Kv1.2 and Kv1.6, Kv1.1 forms the D-current. The D-current has an important role in controlling the delay of spikes, their timing and patterning [11, 30, 35], all important factors for neuronal network plasticity. The D-current can be inhibited by DTX. In our hippocampal cultures, OUL35 occluded DTX sensitivity (Fig. 6c), demonstrating that OUL35 indeed affected the D-current. This effect on the D-current also argues that OUL35 affected Kv1.1α or a closely related Kv1α subunit. A relatively long delay of the first spike is indicative of the D-type K^+^ current [14, 35]. In our experiments, the delay of the first spike evoked at the rheobase was highly variable (Fig. 6a), precluding firm conclusions. The first spike had a mean delay of 89.9 ± 67.6 ms (mean ± SD, *n* = 13) in control neurons and of 196.9 ± 132.1 ms (*n* = 9; *p* = 0.03) in OUL35-treated neurons. A longer delay is unexpected for a reduced D-current [30, 35]. In addition to a decrease in rheobase, an increase in gain is expected for an inhibition of the D-current [14]. We provisionally analyzed the gain as the linear fit of the spike number as a function of current pulses from 10 to 30 pA (Fig. 6b). There was a slight increase in gain in OUL35 treated neurons (0.39 ± 0.12 vs. 0.27 ± 0.08 spikes/pA; *n* = 8), which was not significant, however (*p* = 0.42). The gain was also not significantly increased by compound 20 (0.24 ± 0.18 vs. 0.08 ± 0.06 spikes/pA; *n* = 6–8; *p* = 0.38). Thus, the preliminary analysis neither of the delay to first spike nor of the gain revealed an additional indication for a reduction of the D-current.

Besides PKCδ, ARTD10 is expected to have several other targets in hippocampal neurons [7]. Moreover, PKCδ also has pleiotropic effects and many targets. Therefore, it is expected that OUL35 has inherently complex effects in hippocampal cultures. In addition, chronic treatment (3 days) with OUL35 might elicit homeostatic mechanisms in neurons that further complicate the outcome. The results obtained in neurons, thus, have to be interpreted with caution.

## Conclusions

Our study establishes PKCδ as a new substrate for MARylation by ARTD10. Moreover, it revealed unanticipated, striking effects of pharmacological blockade of ARTD10 on Kv1.1 channels and neuronal excitability, suggesting that MARylation by ARTD10 controls neuronal excitability.

## Methods

### cDNAs

KCNA1 and KCNB1.1 in pGEM were a kind gift of Dr. O. Pongs (Hamburg, Germany). Before transfection, they were cloned in vector pCDNA3. PKCδ CAT and PKCδ DN were contained in vector pHACE and were received from Addgene (Addgene plasmid 16,389 and 16,388, respectively); they were provided by Bernard Weinstein [29]. PKCδ-CAT contains only the catalytic kinase domain without the regulatory domain; PKCδ-DN contains a point mutation at the ATP binding site. Cloning of GST-ARTD10 catalytic domain, pEVRFO-HA-ARTD10 and pEVRFO-HA-ARTD10-G888W, has been described before [5]. pWZL-Neo-Myr-FLAG-PKCδ (Addgene plasmid 20,603; Boehm, 2007) was used to create pDONR/zeo-PKCδ and pDEST17-PKCδ, and pEGFP-PKCδ via the GateWay cloning system. The primers used to amplify PKCδ from the Addgene plasmid were PKCδ_attB1 (GGGGACAAGTTTGTACAAAAAAGCAGGCTCGatggcgccgttcctgcgc) and PKCδ_attB2 (GGGGACCACTTTGTACAAGAAAGCTGGGTC TCAtcaatcttccaggaggtg).

### ADP-ribosylation assays

The His_6_- and GST-tagged fusion proteins were expressed in *E*. *coli* strain BL-21. GST-fusion proteins were purified using glutathione-sepharose, whereas His_6_-PRKCD was purified using TALON metal affinity resin according to standard protocols. One microgram of purified GST-ARTD10 catalytic domain and 0.5–1 μg of purified substrate protein (His_6_-PKCδ) were included in the ADP-ribosylation reactions. ADP-ribosylation assays were routinely carried out in a total volume of 30 μl reaction buffer (50 mM Tris-HCl, pH 8.0; 0.2 mM TCEP; 5 mM MgCl_2_) containing 50 μM β-NAD^+^ (Sigma) and 1 μM Ci [^32^P]-γ-NAD^+^ (Amersham Biosciences) at 30 °C for 30 min. Reactions were stopped by adding SDS sample buffer and were subsequently boiled and separated on SDS-PAGE. To visualize proteins, gels were stained using Coomassie brilliant blue before exposure to X-ray films. Incorporated radioactivity was analyzed by exposure of the dried gel to X-ray film.

### Cell culture and transfection

T-REx HeLa FRT cells (Thermo Fisher Scientific) stably transfected with ARTD10-WT or ARTD10-G888W [26] were grown in DMEM (PAN-Biotech) supplemented with 10% FBS, 10 μg/ml blasticidin, and 75 μg/ml hygromycin B; control HeLa FRT cells were supplemented with 10 μg/ml blasticidin and 75 μg/ml Zeocin. Cells were grown at 37 °C in a humidified atmosphere with 5% CO_2_. Using Lipofectamine™ 2000 (Thermo Fisher Scientific), cells were co-transfected with either rat KCNA1 or rat KCNA1-S446A together with rat KCNAB1. In some cases, PKCδ-CAT or PKCδ-DN was also co-transfected. Expression of ARTD10 and ARTD10-G888W was induced the second day after transfection by the addition of doxycycline (1 μg/ml). Cells were examined 18 to 42 h after induction of ARTD10.

HEK293 cells were grown in DMEM (Gibco) supplemented with 10% FCS and 1 mM sodium pyruvate (Gibco). They were transiently transfected with plasmids encoding EGFP-PKCδ, HA-ARTD10, or the catalytically inactive variant HA-ARTD10-G888W using the calcium phosphate precipitation technique.

Hippocampal neurons were prepared from postnatal mice on day 0 to 2 after birth. Hippocampi from 4 to 10 pups were dissected in ice-cold HBSS (Thermo Fisher Scientific) and digested with 2 μg/ml DNaseI and 50 mg/ml Trypsin in HHGN dissection solution (1 × HBSS, 2.5 mM HEPES, 35 mM glucose, pH 7.4) for 20 min. After washing three times with HBSS, the tissue was triturated with 2 μg/ml DNaseI in BME (+) Earle’s (−) Glutamine solution (Thermo Fisher Scientific). After centrifugation at 200*g* (4 °C) for 5 min, the pellet was resuspended in Neurobasal medium supplemented with 10% (v/v) heat inactivated horse serum and 0.5 mM L-Glutamine. The neurons were plated at a density of 5 × 10^4^/well in 24-well dishes on 35-mm poly-l-ornithine- and laminin- (10 μg/ml) coated cover slips. On the next day, half of the medium was replaced with growth medium (Neurobasal medium supplemented with 1 × B27). On day 7 of culture, the cells were treated with 3 μM OUL35, compound 20, or DMSO as a control. Inhibitors were renewed every 2 days, and the cells were patched on day 9 to day 12.

### In cell MARylation of PKCδ

HEK293 cells co-expressing EGFP-PKCδ and HA-ARTD10-WT were treated with 3 μM OUL35 or the appropriate volume of DMSO as vehicle control for 16 h. Forty-eight hours post transfection, cells were lysed in RIPA buffer (10 mM Tris, pH 7.4; 150 mM NaCl; 1% NP-40; 1% DOC; 0.1% SDS; protease inhibitor cocktail, Sigma) including 10 μM Olaparib (Selleckchem) and the lysates were cleared at 4 °C for 30 min. EGFP-PKCδ was immunoprecipitated with 5 μl of GFP-TRAP magnetic agarose beads (Chromotek) at 4 °C for 1 h. Afterwards, the beads were washed three times in RIPA buffer. Samples were fractionated by SDS-PAGE and transferred to nitrocellulose membranes to visualize MARylation using an anti-PAR/MAR antibody (Cell Signaling Technology, #83732). The individual proteins were analyzed using anti-GFP antibodies (Rockland, mouse monoclonal 600-301-215 M) to visualize EGFP-PKCδ, anti-α-Tubulin (Sigma, #T5168), anti-ARTD10 [6], goat-anti-rabbit-HRP (Jackson Immunoresearch, #111-035-144), and goat-anti-mouse-HRP (Jackson Immunoresearch, #115-036-068).

### Immunoblotting

Cells were lysed with RIPA buffer (25 mM Tris-Cl pH 7.6, 150 mM NaCl, 1% Triton-X-100, 0.1% SDS, 1% sodium deoxycholate, 1% PMSF, and 1% proteinase inhibitor cocktail; Roche) and samples were quantified using a protein assay (Micro BCA; ThermoFisher Scientific). The same amount of protein was separated using SDS-PAGE (10%) and the gel scanned with a Typhoon 9410 Gel and Blot Imager (GE healthcare life sciences).

For immunoblots, proteins were transferred to PVDF membranes (Roche, Mannheim, Germany), and the membrane was blocked for 1 h at RT in 5% non-fat milk in TBS-T (137 mM NaCl, 2.7 mM KCl, 25 mM Tris, 0.1% Tween-20), and probed overnight at 4 °C with the following primary antibodies: rabbit polyclonal anti-Kv1.1 (Alomone Labs, # APC-161 and APC-009), rabbit polyclonal anti-PKCδ (Santa Cruz, # sc-937), rabbit polyclonal anti-phospho-PKCδ (Tyr311) (Cell Signaling, # 2055), rat monoclonal anti-ARTD10 (Merck, #5H11), or mouse monoclonal anti-acetylated tubulin (Sigma-Aldrich, # T7451). Blots were visualized using secondary HRP-conjugated anti-rabbit or anti-mouse antibodies and SuperSignal West Pico or Femto PLUS Chemiluminescent Substrate (ThermoFisher Scientific). The data was analyzed with ImageJ.

### Patch clamping

Coverslips containing HeLa cells or hippocampal neurons were mounted in a perfused bath on the stage of an inverted microscope (IX71, Olympus, Chromaphor) and kept at room temperature. For HeLa cells, the bath solution contained (in mM): 128 NaCl, 5.4 KCl, 10 HEPES, 1 MgCl_2_, 2 CaCl_2_, and 5.5 glucose; pH was adjusted to 7.4. Patch pipettes (4–6 MΩ) were filled with a solution containing (in mM) 121 KCl, 10 NaCl, 10 HEPES, 5 EGTA, and 2 MgCl_2_; pH was adjusted to 7.2. For hippocampal neurons, bath solution contained (in mM) 140 NaCl, 3.5 KCl, 10 HEPES, 2.2 CaCl_2_, 2 MgSO_4_, 1.25 NaH_2_PO_4_, 0.4 KH_2_PO_4_, and 10 glucose; pH was adjusted to 7.3. Patch pipettes contained (in mM) 154 K-gluconate, 6 NaCl, 10 HEPES, 1 EGTA, 2 MgCl_2_, 0.85 CaCl_2_, and 10 glucose; pH was adjusted to 7.3. Patch-clamp recordings were performed in the whole-cell configuration at room temperature. Currents and voltages were recorded using a patch-clamp amplifier (Axopatch 200B; Axon Instruments), an Axon-CNS digitizer (Digidata 1440A), and Clampex 10.3 software (Molecular Devices). Data was filtered at 1 kHz with a low-pass filter and stored continuously on a computer hard disc; it was analyzed using pCLAMP software (Molecular Devices).

For voltage-clamp, the membrane voltage was clamped to − 80 mV and depolarized to 40 mV for 200 ms, and data was sampled at a rate of 4 kHz. For voltage-clamp of hippocampal neurons, voltage-dependent Na^+^ channels were blocked by addition of 0.5 μM TTX to the bath. For current-clamp of hippocampal neurons, 0.1 μg/μl nystatin was added to the pipette solution and the membrane current was clamped to 0 pA for the gap free protocol, and for measurement of the rheobase, ten long (1 s) depolarizing current pulses (increments of 1, 2, 5 or 10 pA) were delivered; data was sampled at a rate of 20 kHz. OUL35 (3 μM) was dissolved in DMSO and culture medium containing DMSO (0.03%) served as a control. To block D-type K^+^ currents, 100 nM DTX was applied through perfusion; currents and voltages were recorded from the same neuron before and 5 min after DTX application.

### Data analysis and statistics

Transient expression of Kv1.1α alone induced robust, non-inactivating K^+^ currents. Additional co-expression of the β subunit conferred a fast inactivating component to these currents. The relative proportions of the inactivating and the non-inactivating component were variable between different batches of transfected cells. We quantified the proportion of the non-inactivating component by building the ratio of the current amplitude at the end of a 200-ms pulse to + 40 mV (*I*_steady-state_) to the peak current at the beginning of this pulse (*I*_peak_). Each data point represents a different cell. In experiments with compound 20-treated hippocampal neurons, the experimenter was blinded to the condition (compound 20 vs. DMSO control). Data are reported as mean ± SEM. Statistical analyses were conducted with Prism 7.0 (GraphPad Software, San Diego, USA). Statistical tests used are reported in the figure legends. We assumed normal distribution and equal variance for each dataset unless otherwise noted, but normal distribution was not formally tested. Two groups were compared using two-tailed paired or unpaired Student’s *t* test, as appropriate. When more than two groups were compared, a one-way analysis of variance (ANOVA) was used followed by Tukey’s multiple comparisons test. *P* ≤ 0.05 was considered as significant. Fisher’s exact test (https://www.socscistatistics.com/) was used to analyze spontaneous AP produced by hippocampal neurons.

## Data Availability

All data generated or analyzed during this study are included in this published article.
